# Brief Web-Based Intervention for Depression: Randomized Controlled Trial on Behavioral Activation

**DOI:** 10.2196/15312

**Published:** 2020-03-26

**Authors:** Lena Jelinek, Sönke Arlt, Steffen Moritz, Johanna Schröder, Stefan Westermann, Barbara Cludius

**Affiliations:** 1 Department of Psychiatry and Psychotherapy University Medical Center Hamburg-Eppendorf Hamburg Germany; 2 Department of Psychiatry and Psychotherapy Evangelical Hospital Alsterdorf Hamburg Germany; 3 Institute for Sex Research, Sexual Medicine and Forensic Psychiatry University Medical Center Hamburg-Eppendorf Hamburg Germany

**Keywords:** affective disorders, depressive symptoms, brief psychotherapy, internet, world wide web

## Abstract

**Background:**

Web-based interventions have been shown to be effective for the treatment of depression. However, interventions are often complex and include a variety of elements, making it difficult to identify the most effective component(s).

**Objective:**

The aim of this pilot study was to shed light on mechanisms in the online treatment of depression by comparing a single-module, fully automated intervention for depression (internet-based behavioral activation [iBA]) to a nonoverlapping active control intervention and a nonactive control group.

**Methods:**

We assessed 104 people with at least mild depressive symptoms (Patient Health Questionnaire-9, >4) via the internet at baseline (t_0_) and 2 weeks (t_1_) and 4 weeks (t_2_) later. After the t_0_ assessment, participants were randomly allocated to one of three groups: (1) iBA (n=37), (2) active control using a brief internet-based mindfulness intervention (iMBI, n=32), or (3) care as usual (CAU, n=35). The primary outcome was improvement in depressive symptoms, as measured using the Patient Health Questionnaire-9. Secondary parameters included changes in activity, dysfunctional attitudes, and quality of life

**Results:**

While groups did not differ regarding the change in depression from t_0_ to t_1_ (η_p_^2^=.007, *P*=.746) or t_0_ to t_2_ (η_p_^2^=.008, *P*=.735), iBA was associated with a larger decrease in dysfunctional attitudes from t_0_ to t_2_ in comparison to CAU (η_p_^2^=.053, *P*=.04) and a larger increase in activity from t_0_ to t_1_ than the pooled control groups (η_p_^2^=.060, *P*=.02). A change in depression from t_0_ to t_2_ was mediated by a change in activity from t_0_ to t_1_. At t_1_, 22% (6/27) of the participants in the iBA group and 12% (3/25) of the participants in the iMBI group indicated that they did not use the intervention.

**Conclusions:**

Although we did not find support for the short-term efficacy of the single-module iBA regarding depression, long-term effects are still conceivable, potentially initiated by changes in secondary outcomes. Future studies should use a longer intervention and follow-up interval.

**Trial Registration:**

DKRS (#DRKS00011562)

## Introduction

Major depressive disorder (MDD) is a disabling disorder that affects millions of people worldwide. Besides the traditional interventions of pharmacotherapy and face-to-face psychotherapy, psychological online interventions (POI) have been established to treat patients with MDD over the last 20 years. Evidence for the efficacy of POI in the treatment of depression is accumulating [1-4], with a recent meta-analysis reporting a pooled effect size of *g*=−0.90 for interventions using an online or mobile setting, in comparison to the use of a waitlist, for adults with a confirmed diagnosis of unipolar depression [5]. The effects are similar to face-to-face treatment [6]. For subthreshold depression, however, a meta-analysis by Zhou et al [7] only showed a small effect, with a standardized mean difference of –0.28.

POIs for depression vary substantially with regard to content; level of guidance/support (guided, unguided, or self-guided [8,9]); duration, with most interventions lasting between 4 and 12 weeks [5]; the degree to which patients can tailor or self-select specific intervention components; and the degree to which interventions focus on a single stand-alone technique or incorporate multiple therapeutic techniques (eg, cognitive behavioral-based interventions for depression usually include psychoeducation, behavior monitoring, cognitive restructuring, and behavioral activation). These differences make it difficult to investigate the mechanisms of change and identify which techniques are the most important for change, as demonstrated for depression [10]. This is often due to the methodological characteristics in the general design of psychotherapeutic trials that can complicate analyses by focusing on the overall effect [11,12], obstructing the larger aim of expanding existing knowledge about change processes and further refining interventions.

Brief interventions reduced to their essentials are a potential alternative to investigate mechanisms of change. A first attempt to evaluate a focused single-module brief intervention for depression was made by Lüdtke et al [13]. However, cognitive restructuring and behavior activation were combined in one module, preventing a thorough investigation of the mechanisms of change.

Generally, cognitive restructuring and behavior activation are two standard interventions in cognitive behavioral therapy (CBT) for depression. Most commonly, treatment starts with interventions aimed at increasing the level of (positive) activities in patients by, for example, monitoring daily activities and mood, scheduling activities, and coping with problems. This “behavioral activation” (BA) has been adapted from behavior therapy for depression, which is also referred to as Lewinsohn’s model [14], and incorporated in CBT [15] to increase patients’ access to positive reinforcement through positive activities. For a more thorough history of BA, we would like to refer to Quigley and Dobson [16]. In 1996, Jacobson et al [17] showed that BA improved depression and dysfunctional beliefs similar to a complete CBT program, fueling the refinement of theory and practice in the use of BA as a stand-alone treatment. Refined BA models were subsequently introduced by Martell et al [18] and Lejuez et al [19], among others.

As a stand-alone intervention for depression, BA has been found similarly effective to CBT in face-to-face treatment. It is regarded as particularly cost-effective [20]. The Canadian Network for Mood and Anxiety Treatments even recommends BA as a first-line treatment for depression [21]. Still, as with other interventions, the mechanisms of actions are unclear for BA [22].

Regarding internet-delivered BA (iBA), a recent meta-analysis by Huguet et al [23] supports its effectiveness, although conclusions were compromised by the low quality of many of the studies. In this meta-analysis, iBA interventions lasted between 6 and 17 weeks. One of the studies [24] compared BA to physical activity and a waitlist control, and depression decreased more in the treatment groups than in the waitlist control group. There was no relationship between the number of modules used and the decrease in symptoms. Thus, it remains unclear how many modules or sessions of iBA are needed to achieve a response.

Another intervention that is often used in combination with CBT in depression is mindfulness (ie, mindfulness-based cognitive therapy [MBCT]). In face-to-face treatment, some evidence suggests the effectiveness of MBCT for depression [25]. When mindfulness-based interventions, not limited to MBCT, are administered online, meta-analyses have shown small to moderate effects with a Hedge *g* between 0.29 [26] and 0.61 [27], representing a general decrease in depressive symptoms. However, the effects were not significant in the meta-analysis by Sevilla-Llewellyn-Jones et al [27] when only participants with depression were considered (*g*=−0.69, 95% CI −1.694 to −0.313, *P*=.19).

To the best of our knowledge, there is only one internet-delivered randomized controlled trial [28] that compared BA and a mindfulness-based intervention as an active control group. Smartphone-administered BA and mindfulness interventions were compared over a treatment period of 8 weeks. Overall, both interventions showed a similar effect on depression. However, whereas BA was more effective for participants with a higher severity of depression at baseline (ie, Patient Health Questionnaire-9 [PHQ-9] total score ≥10 and a diagnosis of moderate major depression), mindfulness was more effective for those with lower symptom severity.

The aim of this study was to use a maximum-focused approach to assess the short-term effects of brief Web-based interventions and the potential mechanisms of change. We investigated a brief iBA module compared with an active control in the form of internet-delivered mindfulness (iMBI) and a nonactive control in the form of a waitlist with full access to care as usual (CAU). With regard to overlap in psychoeducation content and specific interventions such as the techniques and skills taught to the participants, we regarded the overlap between iBA and iMBI as minimal. We assessed participants at baseline (t_0_) and then 2 weeks (t_1_) and 4 weeks (t_2_) later. The primary outcome was the improvement in depressive symptoms, as assessed with the PHQ-9, from t_0_ to t_2_. We chose the 4-week interval, instead of the 2-week interval, for the primary outcome to optimize the comparability of our results to other studies, which often use longer intervals of 8-9 weeks [5], and to investigate lasting change in depression. Secondary parameters included change from t_0_ to t_1_ in depressive symptoms, as measured with the PHQ-9; change in activity, as measured with the Behavioral Activation for Depression Scale (BADS); mindfulness, as measured with the Kentucky Inventory of Mindfulness Skills (KIMS); dysfunctional cognitive biases, as measured with the Dysfunctional Attitude Scale (DAS); and subjective quality of life, as measured with the World Health Organization Quality of Life (WHOQOL). We wanted to (1) pilot test the efficacy of the brief iBA module and (2) explore the mechanisms of change. Based on Ly et al [28], we expected a reduction in both the primary and secondary outcomes in favor of the experimental intervention (iBA), when compared with the active (iMBI) and nonactive (CAU) control groups. Moreover, we conducted mediation analyses to investigate the mechanisms of change. We expected that an improvement in depressive symptoms at t_2_ would be mediated by change in activation (BADS) between t_0_ and t_1_. To further explore the mechanisms of change, we also included change in mindfulness using the KIMS, which mediates changes in MBCT [29], and dysfunctional cognitive biases using the DAS, which has been generally been associated with change in depression [30], as mediators in the exploratory analyses.

## Methods

### Design

We performed a parallel randomized controlled trial. Participants were assessed using the online survey program EFS Survey developed by Questback (Oslo, Norway). Following the assessment at t_0_, participants were randomly allocated to iBA, which included CAU; iMBI, which also included CAU; or CAU only. CAU included full access to treatment as usual. Participants were reassessed after 2 weeks (t_1_) and 4 weeks (t_2_). All participants provided electronic informed consent. The trial was approved by the Ethics Committee of the German Psychological Association (LJ032018_amd_102016) and was preregistered with the German Clinical Trials Register (DRKS; #DRKS00011562). Some changes were made after the trial was registered. The aim of the original study was to recruit treatment-seeking outpatients through medical staff at the hospital. As this led to very low participation, we decided to recruit through online advertisements instead. Therefore, depressive symptoms according to the PHQ-9 instead of a depressive disorder as diagnosed by a therapist/practitioner was used as an inclusion criterion.

### Participants and Procedures

Recruitment took place between April 18, 2018, and May 27, 2018, via a Google AdWords campaign. The link to the study was also sent to participants from previous studies who had provided written consent to be contacted again via email. Inclusion criteria were depressive symptoms according to self-report (PHQ-9 total score >4 indicating mild depression), age between 18 and 65 years, internet access, internet literacy, sufficient German language skills, and informed consent, including the willingness to participate in 3 online assessments and a 2-week intervention. Exclusion criteria were lifetime psychotic or manic symptoms and suicidality as indicated by a score >2 on the Beck Depression Inventory II suicide item. In case of exclusion due to suicidal tendencies, help was provided in the form of emergency phone numbers and Web addresses of health services. Group allocation was performed by a person who did not possess any other information on the respective participant. Participants were randomized at t_0_ to one of the 3 groups, with an allocation ratio of 1:1:1 based on a fixed randomization plan conducted by a statistician. This has been referred to as centralized assignment [31]. After randomization, participants were informed about their allocation via email. This email gave participants assigned to the iBA and iMBI groups a link, code, and password to access the appropriate Web-based treatment. They could use the treatment at the times, pace, and frequency they chose to meet their needs. All participants in the intention-to-treat (ITT) group were sent a link for re-assessment at 2 and 4 weeks later. To increase the retention rate, participants were reminded every 2 days to complete the survey (up to 3 times in total). After completion of the 4-week assessment, all participants received the links, codes, and passwords to access both treatments.

**Figure 1:**
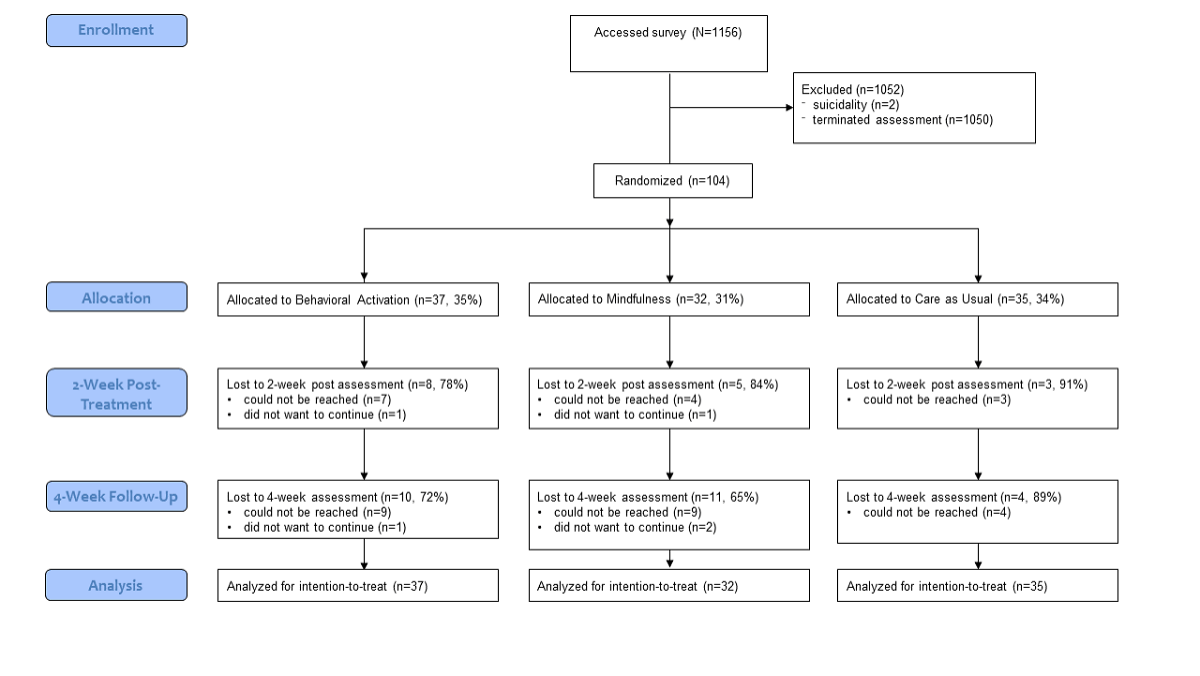
Flowchart of participation in the study.

In total, 1156 people accessed the survey (see Figure 1). The majority did not finish the baseline survey (n=1050), and the assessment was automatically terminated for 2 participants because they met exclusion criteria (n=2 suicidality; see exclusion criteria) leaving 104 participants for the ITT group.

### Interventions

Both Web-based interventions were unguided. All information on how to use the interventions was provided within each program; we did not provide any additional information, including in the reminders. The interventions consisted of psychoeducational information as well as worksheets. Worksheets could be saved on the computer or printed for daily use. For iMBI, that also included audio files. Participants were also allowed to log into the module repeatedly to access clean worksheets or to reread the (psychoeducation) information provided.

#### Experimental Intervention: Internet-Based Behavioral Activation

**Figure 2:**
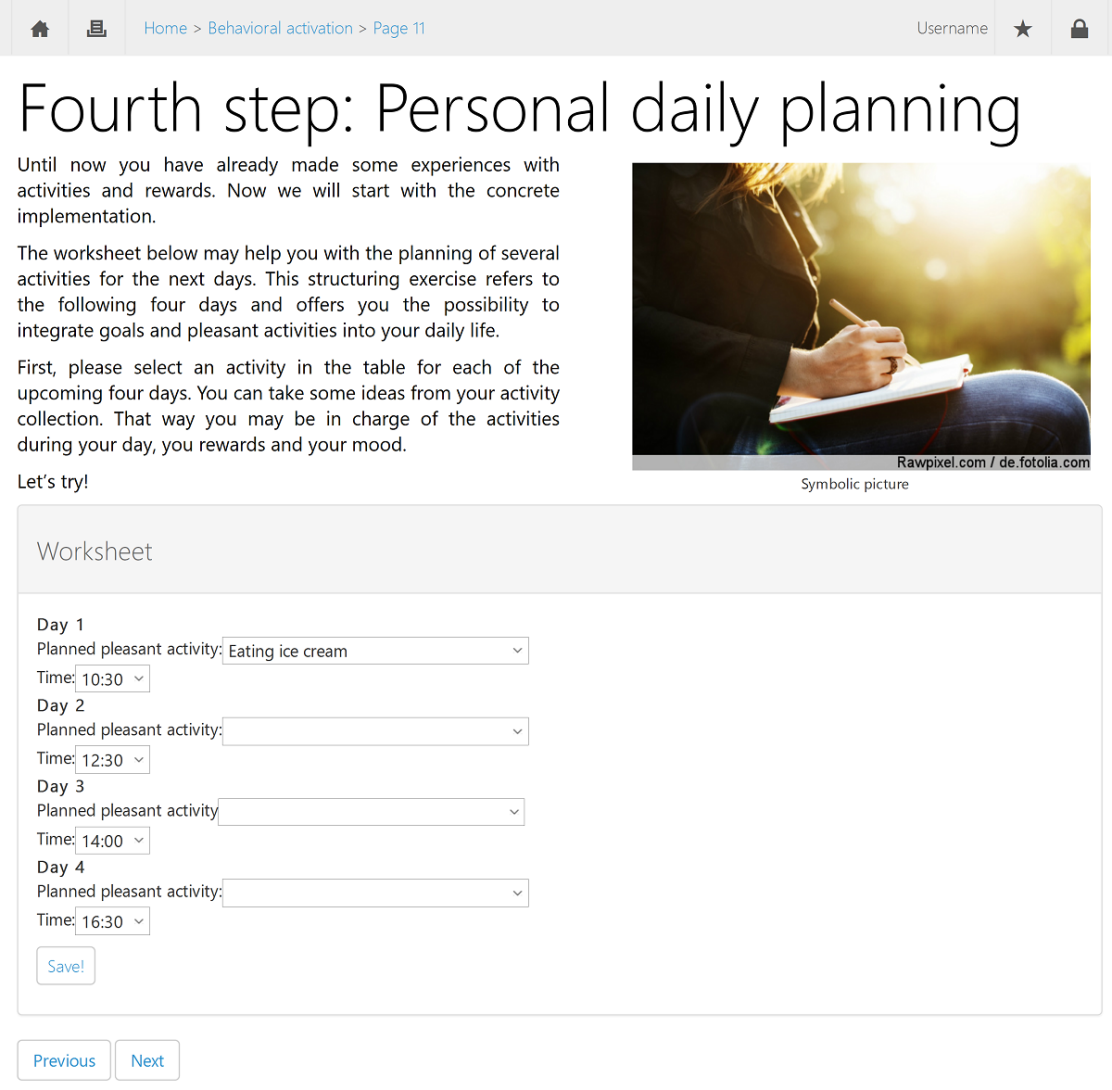
Screenshot of the experimental, internet-based behavioral activation intervention (translated).

iBA is a Web-based intervention that focuses on the development of behavioral activities. We used a version based on the German version of the manual by Martell et al [32]. The intervention starts with psychoeducation on the interplay between mood and behavior in depression. Then, participants learn how to monitor their mood and daily activities, sensibly plan activities in their daily schedule, and anticipate problems that may occur when they try to perform the planned activities. Most of the information is accompanied by worksheets that can be filled out and saved on a computer or printed for daily use. Depending on the individual’s reading rate and personal processing time, it takes about 60 minutes to become familiar with the information in the module. However, participants are advised to take their time and use the program daily, including completing the work sheets and performing the planned activities. For a screenshot of the intervention in a Web browser, see Figure 2.

#### Active Control Group: Internet-Based Mindfulness

iMBI is a Web-based intervention introducing and teaching mindfulness practice. We used a version based on the German version of the mindfulness manual by Segal et al [33]. It starts with an introduction into the concept of mindfulness (eg, the “automatic pilot”) and an explanation of how mindfulness can help with depression. Then, participants independently carry out mindfulness exercises, starting with a short exercise on mindful listening. They use worksheets to detect situations in which they are not mindful (on automatic pilot), how to mindfully deal with disturbing thoughts and feelings (“thoughts are not facts”), and how to enhance self-care through mindfulness. In the module, a variety of audio files are provided to optimize mindfulness practice: breathing exercise (5:50 min), body scan (16:15 min), and inner smile (4:40 min). Depending on the reading rate and personal processing time, it takes about 60 minutes to read the content of the module and become familiar with the worksheets and exercises. Similar to the iBA intervention, participants are advised to use the exercises and worksheets in their daily life and to use the program repeatedly. In this study, to keep the practice time similar to that of the iBA group, participants were asked to practice only 1 of the 3 mindfulness exercises repeatedly over the following week.

#### Care as Usual Only

CAU included full access to treatment as usual provided by the German health care system. As an incentive, participants received both Web-based interventions (iBA and iMBI) for their personal use after finishing the 4-week assessment.

### Measures

The primary outcome was improvement in depressive symptoms, as assessed with the PHQ-9, from t_0_ to t_2_. Secondary outcomes included changes from t_0_ to t_1_ in depressive symptoms (PHQ-9), activity (BADS), mindfulness (KIMS), dysfunctional attitudes (DAS), and subjective quality of life (WHOQOL). Negative effects were assessed by calculating the reliable change index for clinically significant deterioration in the PHQ-9 from t_0_ to t_1_ [34].

#### Primary Outcome

The severity of depression was measured using the German version of the PHQ-9 [35]. The PHQ-9 represents the depression module of the PHQ-D and uses 9 items rated on a 4-point Likert scale. Good validity and reliability have been reported [36,37].

#### Secondary Outcomes

The German version of the BADS [38] was used to assess levels of behavioral activation. It includes 25 items summarized as a total score. Internal consistency and test-retest reliability are considered acceptable [39]. Dispositional mindfulness was assessed with the German version of the 20-item version of the KIMS [40]. The psychometric properties of the KIMS, including sensitivity of change, are considered good [41]. The German 18-item version of the DAS form B (DAS-18B) was used to assess dysfunctional beliefs. Items are rated on a 7-point Likert scale. Reliability and validity of the scale are good [42]. Quality of life was assessed with the global item of the German version of the WHOQOL-BREF [43].

### Data Analysis

SPSS 25.0 (IBM Corp, Armonk, NY) software was used for all analyses. We conducted complete-cases (CC) as well as ITT analyses. CC analyses were based on data from participants who were assessed at all 3 assessment points. For ITT analyses, which included all randomized participants, missing data were imputed by multiple imputations based on the assumption that data were missing at random, conditional on information on treatment, sex, age, and all relevant outcomes across the 3 assessment time points. We created 100 imputed datasets.

To investigate efficacy, we conducted analyses of covariance (ANCOVA) with treatment (iBA, iMBI, CAU) as the independent factor, the baseline level of the respective outcome as the covariate, and the level of the outcome at t_1_ and t_2_ as the dependent variable. To follow up on group differences, control groups were pooled to compare the experimental iBA group with the control iMBI and CAU groups. For post-hoc tests at the individual group level, we used uncorrected *t* tests. Effect sizes for ANCOVAs are reported according to Kinnear and Gray [44], with η_p_^2^≈.01, η_p_^2^≈.06, and η_p_^2^≈.14 corresponding to small, medium, and large effects, respectively. We also calculated Cohen *d* as an effect size for change in outcomes over time within each of the groups, with │*d*│≈.2, │*d*│≈.5, and │*d*│≈.8 corresponding to small, medium, and large effects, respectively.

**Figure 3:**
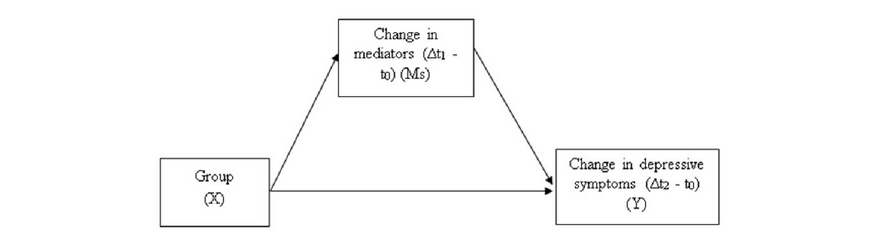
Differences between baseline (t_0_) and the 2-week post-treatment (t_1_) and the 4-week follow-up (t_2_) assessments were calculated as standardized residualized change scores. Group was defined as experimental (internet-based behavioral activation) vs the pooled control groups (internet-based mindfulness intervention and care as usual). Mediators were measured using the Behavioral Activation for Depression Scale, Kentucky Inventory of Mindfulness Skills, and Dysfunctional Attitude Scale (German 18-item version of form B). Depressive symptoms were measured using the Patient Health Questionnaire-9.

To investigate the mechanisms of change involved in the interventions, we conducted mediation analyses [45]. We coded iBA as 1 and pooled the control groups (iMBI and CAU) and coded them as 0, with the treatment effect referring to the effects of iBA above and beyond the pooled control groups. We computed standardized, residualized change scores for change in PHQ-9 from t_0_ to t_2_ and for change in mediators (BADS, KIMS, DAS) from t_0_ to t_1_ (see Figure 3). The mediation analyses were performed using the SPSS macro PROCESS developed by Hayes (version v3.1) [46] and met all the criteria defined by Kraemer et al [47] for the use of mediators within a randomized controlled trial. We bootstrapped the results 5000 times to correct for potential biases of nonnormality in the sample. For mediation analyses, we used a different strategy to impute missing data, that is, the expectation-maximization algorithm. This was necessary because multiple imputation data sets cannot be used in PROCESS, which requires a single data set. The mediation hypothesis is confirmed when the effect range (lower limit of the 95% CI [LLCI] to upper limit of the 95% CI [ULCI]) does not include zero.

## Results

### Demographic Characteristics

Table 1 shows the demographic characteristics of the 3 groups. The participants were mostly (80/104, 76.9%) female, were in their mid-forties on average, and had almost 12 years of formal education. Previous psychological treatment experiences, including internet interventions and use of self-help books, were high but similar between groups (*P*>.40). Randomization was successful, with no significant differences in the demographic characteristics between groups.

**Table 1:** Baseline demographics.

Variable		iBA^a^ (n=37)	iMBI^b^ (n=32)	CAU^c^ (n=35)	Total sample (N=104)	Statistic	*P* value
Age (years), mean (SD)		45.84 (10.29)	44.91 (10.98)	47.80 (9.54)	46.21 (10.24)	*F*(2,101)=0.702	.50
**Sex, n (%)**							
	Female	32 (86)	25 (78)	23 (66)	80 (76.9)	χ²_2_=4.409	.11
	Male	5 (14)	7 (22)	12 (34)	24 (23.1)		
Years of formal education, mean (SD)		11.97 (1.46)	11.84 (1.37)	11.62 (1.50)	11.81 (1.44)	*F*(2,101)=0.517	.60
Number of psychotherapy sessions, mean (SD)		92.27 (93.07)	92.00 (122.12)	74.83 (66.96)	86.32 (95.14)	*F*(2,101)=0.380	.69
Number of hospitalizations, mean (SD)		1.03 (1.26)	1.38 (2.14)	1.40 (2.20)	1.26 (1.89)	*F*(2,101)=0.434	.65
Number of self-help books, mean (SD)		6.35 (6.83)	10.72 (26.51)	7.03 (6.77)	7.92 (15.70)	*F*(2,101)=0.745	.48
Number of online interventions, mean (SD)		1.19 (1.51)	0.84 (1.11)	1.00 (1.14)	1.02 (1.27)	*F*(2,101)=0.531	.53

^a^iBA: internet-based behavioral activation.

^b^iMBI: internet-based mindfulness intervention.

^c^CAU: care as usual.

### Adherence and Completion

At t_1_, 6 participants in the iBA group (6/27, 22%) and 3 participants in the iMBI group (3/25, 12%) indicated that they had not used the respective online module at all; due to technical difficulties, 2 participants in the iBA group and 2 participants in the iMBI group were not asked whether they used the respective intervention. In the ITT group (N=104), 88 participants (85%) completed the t_1_ assessment, and 79 (76%) completed the t_2_ assessment.

### Outcome Analyses

The assessment values for all 3 assessment points for the CC group are displayed in Table 2. Regarding the primary outcome (PHQ-9), the proposed superiority of iBA was confirmed in neither the CC sample nor the ITT sample (Tables 3 and 4). In the iBA group, however, depression decreased with a medium effect from t_0_ to t_2_ (*d*=−0.53).

**Table 2:** Primary and secondary outcome measures in the complete-cases group.

	Baseline (t_0_), mean (SD)			2-weeks post-treatment (t_1_), mean (SD)			4-week follow-up (t_2_), mean (SD)		
	iBA^a^ (n=37)	iMBI^b^ (n=32)	CAU^c^ (n=35)	iBA (n=29)	iMBI (n=27)	CAU (n=32)	iBA (n=27)	iMBI (n=21)	CAU (n=31)
PHQ-9^d^	10.46 (4.32)	12.50 (5.04)	11.00 (4.27)	9.73 (5.46)	11.37 (6.15)	9.90 (3.14)	8.70 (5.72)	11.57 (5.27)	9.26 (4.14)
BADS^e^	71.97 (18.91)	72.50 (24.62)	73.97 (20.32)	85.76 (22.90)	77.19 (23.95)	78.59 (21.63)	86.85 (27.22)	81.19 (23.84)	84.29 (26.70)
KIMS^f^	117.68 (15.71)	112.78 (17.11)	114.97 (19.11)	119.97 (15.98)	115.67 (17.19)	113.03 (18.22)	119.89 (16.40)	115.24 (19.10)	112.97 (19.18)
DAS-18B^g^	66.68 (19.65)	69.19 (20.47)	69.29 (18.41)	62.14 (14.97)	64.44 (19.72)	67.68 (19.99)	56.44 (20.49)	64.10 (20.62)	67.94 (19.12)
WHOQOL^h^	3.08 (0.68)	2.91 (0.86)	2.94 (0.73)	3.28 (0.88)	3.04 (0.71)	3.12 (0.66)	3.11 (1.01)	3.05 (0.74)	2.94 (0.77)

^a^iBA: internet-based behavioral activation.

^b^iMBI: internet-based mindfulness intervention.

^c^CAU: care as usual.

^d^PHQ-9: Patient Health Questionnaire-9.

^e^BADS: Behavioral Activation for Depression Scale.

^f^KIMS: Kentucky Inventory of Mindfulness Skills.

^g^DAS-18B: German 18-item version of the Dysfunctional Attitude Scale form B.

^h^WHOQOL: World Health Organization Quality of Life Scale (global item).

**Table 3:** Outcome analyses, including the effect sizes for and between-group differences in the outcome measures between the baseline (t_0_) and 2-week post-treatment (t_1_) assessments in the complete-cases group.

	Effect size (*d*)			Between-group differences^a^			
	iBA^b^	iMBI^c^	CAU^d^	Statistic	Effect size (η_p_^2^)	90% CI	*P* value
PHQ-9^e^	–0.23	–0.31	–0.35	*F*(2,84)=0.29	.007	0.000- 0.041	.75 (81^f^)
BADS^g^	0.96	0.25	0.30	*F*(2,84)=2.73	.061	0.000-0.145	.07^h^ (.09^f^)
KIMS^i^	0.27	0.31	–0.19	*F*(2,84)=3.00	.067	0.000-0.152	.06^j^ (.03^f^)
DAS-18B^k^	–0.37	–0.37	–0.14	*F*(2,84)=1.11	.026	0.000-0.088	.34 (.14^f^)
WHOQOL^l^	0.31	0.16	0.26	*F*(2,84)=0.56	.013	0.000-0.061	.57 (.56^f^)

^a^Assessed using analyses of covariance (ANCOVA) with baseline scores as covariates and uncorrected *t* tests for post-hoc tests.

^b^iBA: internet-based behavioral activation.

^c^iMBI: internet-based mindfulness intervention.

^d^CAU: care as usual.

^e^PHQ-9: Patient Health Questionnaire-9.

^f^Intention-to-treat analyses based on multiple imputation.

^g^BADS: Behavioral Activation for Depression Scale.

^h^BA>iMBI.

^i^KIMS: Kentucky Inventory of Mindfulness Skills.

^j^iBA>CAU.

^k^DAS-18B: German 18-item version of the Dysfunctional Attitude Scale form B.

^l^WHOQOL: World Health Organization Quality of Life Scale (global item).

**Table 4:** Outcome analyses, including the effect sizes for and between-group differences in the outcome measures between the baseline (t_0_) and 4-week follow-up (t_2_) assessments in the complete-cases group.

	Effect size (*d*)			Between-group differences^a^				
	iBA^b^	iMBI^c^	CAU^d^		Statistic	Effect size (η_p_^2^)	90% CI	*P* value
PHQ-9^e^	–0.53	–0.24	–0.53		*F*(2,75)=0.31	.008	0.000-0.048	.74 (.99^f^)
BADS^g^	0.98	0.44	0.64		*F*(2,75)=0.27	.007	0.000-0.044	.76 (.52^f^)
KIMS^h^	0.24	0.25	–0.18		*F*(2,75)=1.08	.028	0.000-0.096	.34 (.11^f^)
DAS-18B^i^	−0.87	–0.41	–0.12		*F*(2,75)=3.62	.088	0.004-0.186	.03^j^ (.008^f^)
WHOQOL^k^	0.05	0.18	0		*F*(2,75)=1.07	.028	0.000-0.095	.35 (.59^f^)

^a^Assessed using analyses of covariance (ANCOVA) with baseline scores as covariates and uncorrected *t* tests for post-hoc tests.

^b^iBA: internet-based behavioral activation.

^c^iMBI: internet-based mindfulness intervention.

^d^CAU: care as usual.

^e^PHQ-9: Patient Health Questionnaire-9.

^f^Intention-to-treat analyses based on multiple imputation.

^g^BADS: Behavioral Activation for Depression Scale.

^h^KIMS: Kentucky Inventory of Mindfulness Skills.

^i^DAS-18B: German 18-item version of the Dysfunctional Attitude Scale form B.

^j^iBA>CAU.

^k^WHOQOL: World Health Organization Quality of Life Scale (global item).

For secondary outcomes, a significant medium effect was found for improvement in dysfunctional attitudes (DAS-18B) from t_0_ to t_2_ (CC and ITT analyses). Improvement in the DAS-18B was larger in the iBA group than in the pooled control groups (η_p_^2^=.053, *P*=.04), which was explained by the larger decrease in dysfunctional attitudes (DAS-18B) in the iBA than in the CAU group in the single group comparisons. On trend level, the groups in the CC sample also differed in the improvements in level of activation (BADS) and mindfulness skills (KIMS) from t_0_ to t_1_; the differences were significant in the ITT analyses. When the control groups were pooled, activity increased more in the iBA group than the pooled control groups (η_p_^2^=.060, *P*=.02), but the differences in KIMS were no longer significant (*P*=.11). Single comparisons showed that the increase in KIMS was larger in the iBA than in the CAU group and that the level of activation (BADS) increased in the iBA group more than in the iMBI group. The groups did not differ in the change in quality of life (WHOQOL) over time.

Regarding changes in the secondary outcomes in the iBA group, the effects were large for increase in activity (BADS, d between 0.96 and 0.98), and the decrease in dysfunctional attitudes reached a medium to large effect (*d* between 0.37 and 0.87). See Tables 3 and 4 for more details.

The deterioration rates were similar between the groups (χ²_2_=1.47, *P*=.48), with a mean deterioration rate of 6.8%.

### Mediation

Intercorrelations between all outcome parameters at all assessment points are shown in Table 5.

**Table 5:** Zero-order correlations for the primary and secondary outcomes at baseline (t_0_), 2-week post-treatment (t_1_), and 4-week follow-up (t_2_).

		PHQ-9^a^, t_0_	DAS-18B^b^, t_0_	KIMS^c^, t_0_	BADS^d^, t_0_	PHQ-9, t_1_	DAS-18B, t_1_	KIMS, t_1_	BADS, t_1_	PHQ-9, t_2_	DAS-18B, t_2_	KIMS, t_2_	BADS, t_2_
**PHQ-9, t_0_**													
	r	—^e^	.15	–.23	–.53	.73	.17	–.25	–.46	.70	.15	–.30	–.35
	*P* value^f^	—	—	—	—	—	—	—	—	—	—	—	—
**DAS-18B, t_0_**													
	r	—	—	–.57	–.33	.11	.80	–.54	–.22	.09	.82	–.50	–.28
	*P* value^f^	.134	—	—	—	—	—	—	—	—	—	—	—
**KIMS, t_0_**													
	r	—	—	—	.54	–.27	–.52	.85	.43	–.25	–.49	.83	.41
	*P* value^f^	.017	.001	—	—	—	—	—	—	—	—	—	—
**BADS, t_0_**													
	r	—	—	—	—	–.52	–.38	.46	.71	–.52	–.28	.50	.68
	*P* value^f^	.001	.001	.001	—	—	—	—	—	—	—	—	—
**PHQ-9, t_1_**													
	r	—	—	—	—	—	.23	–.34	–.71	.83	.15	–.35	–.51
	*P* value^f^	.001	.284	.006	.001	—	—	—	—	—	—	—	—
**DAS-18B, t_1_**													
	r	—	—	—	—	—	—	–.58	–.34	.16	.87	–.51	–.33
	*P* value^f^	.085	.001	.001	.001	.019	—	—	—	—	—	—	—
**KIMS, t_1_**													
	r	—	—	—	—	—	—	—	.50	–.26	–.51	.89	.43
	*P* value^f^	.011	.001	.001	.001	.001	.001	—	—	—	—	—	—
**BADS, t_1_**													
	r	—	—	—	—	—	—	—	—	–.61	–.23	.59	.76
	*P* value^f^	.001	.027	.001	.001	.001	.001	.001	—	—	—	—	—
**PHQ−9, t_2_**													
	r	—	—	—	—	—	—	—	—	—	.17	–.37	–.64
	*P* value^f^	.001	.352	.011	.001	.001	.115	.009	.001	—	—	—	—
**DAS-18B, t_2_**													
	r	—	—	—	—	—	—	—	—	—	—	–.53	–.33
	*P* value^f^	.141	.001	.001	.004	.120	.001	.001	.022	.095	—	—	—
**KIMS, t_2_**													
	r	—	—	—	—	—	—	—	—	—	—	—	.55
	*P* value^f^	.002	.001	.001	.001	.001	.001	.001	.001	.001	.001	—	—
**BADS, t_2_**													
	r	—	—	—	—	—	—	—	—	—	—	—	—
	*P* value^f^	.001	.005	.001	.001	.001	.001	.001	.001	.001	.001	.001	—

^a^PHQ-9: Patient Health Questionnaire-9.

^b^DAS-18B: German 18-item version of the Dysfunctional Attitude Scale form B.

^c^BADS: Behavioral Activation for Depression Scale.

^d^KIMS: Kentucky Inventory of Mindfulness Skills.

^e^Not applicable.

^f^two-tailed.

From the 3 potential mediators (changes in BADS, KIMS, and DAS-18B) from t_0_ to t_1_, only the change in BADS acted as a mediator for the change in depression (PHQ-9) from t_0_ to t_2_. Change in BADS was the only significant mediator; the changes in KIMS and DAS-18B did not mediate the change in depression (see Table 6). For the change in depression as measured by BADS, the indirect effect was −.20 (SE 0.09, LLCI=-0.40, ULCI=−0.03), with a remaining nonsignificant direct effect of the treatment of 0.10 (SE 0.20, *P*=.60, LLCI=−0.29, ULCI=0.50).

**Table 6:** Mediation analysis: Effects of treatment (internet-based behavioral activation, coded as 1, vs the pooled control groups, coded as 0) on the mediators and the effects of the mediators (including treatment) on depression, as measured by the Patient Health Questionnaire-9 (PHQ-9).

	Mediators														Outcome				
	DAS-18B^a^				KIMS^b^					BADS^c^					PHQ-9				
	beta	SE	*P* value	beta		SE	*P* value		beta		SE	*P* value		beta			SE	*P* value	
Treatment	–.23	0.20	.255	.32		0.20	.116		.49		0.20	.015		.10			.20	.601	
DAS-18B	—^d^	—	—	—		—	—		—		—	—		.07			.10	.472	
KIMS	—	—	—	—		—	—		—		—	—		.14			.10	.174	
BADS	—	—	—	—		—	—		—		—	—		–.41			.10	<.001	
*R* ^2^	.01	—	—	.02		—		—		.06	—	—			.15		—		—

^a^DAS-18B: German 18-item version of the Dysfunctional Attitude Scale form B.

^b^KIMS: Kentucky Inventory of Mindfulness Skills.

^c^BADS: Behavioral Activation for Depression Scale.

^d^Not applicable.

When we recalculated the mediation analyses to only compare the iBA and iMBI groups with regard to potential mediators, only the indirect effect of the BADS was significant again (DAS-18B: beta=−.02, SE 0.05, LLCI=−0.14, ULCI=0.08; KIMS: beta=.01, SE 0.04, LLCI=−0.07, ULCI=0.11; BADS: beta = −.18, SE 0.10, LLCI=−0.41, ULCI=−0.002; Total: beta=–17, SE 0.09, LLCI=–.36, ULCI=–.01).

## Discussion

The aim of this study was to pilot test the efficacy of iBA, a brief Web-based module using BA, in comparison to active (iMBI) and nonactive (CAU) control interventions and to explore the mechanisms of change.

### Primary Outcome

Contrary to our expectation, the reduction in the primary outcome of depressive symptoms (PHQ-9) was similar between all the groups. Moreover, the response rate and effect size were smaller than in previous studies that showed BA is efficacious in both face-to-face [20] and online [23] interventions. In this study, the iBA group experienced a symptom decrease of 17% and a medium effect of *d*=−0.53 from t_0_ to t_2_, compared with a 7% decrease and *d* of −0.24 in the iMBI group and a 16% decrease and *d* of −0.53 in the CAU group. Thus, reasons for the present null findings need to be discussed.

First, to assess the mechanisms of change, we introduced BA as a single-module, internet-based intervention (iBA). The recent meta-analysis by Huguet et al [23] showed efficacy for iBA; however, 3 of the 9 studies combined iBA with other interventions. Therefore, it is unclear whether the treatment effect was attributable to iBA or another active component. Moreover, not one but several BA sessions were used, covering periods between 6 and 17 weeks. Another important difference between our study and those in the meta-analysis of Huguet et al is we used an unguided, fully automated intervention, as opposed to guided interventions. To the best of our knowledge, Lüdtke et al [13] conducted the only study that used a single-module, unguided internet-based intervention for depression, but it combined behavior activation with cognitive restructuring and also found no effect on depressive symptoms. Although previous studies have shown an effect of unguided interventions on depression [9], the results of our study and that by Lüdtke et al may indicate that iBA cannot be learned without therapist contact or that a single-module approach is insufficient to cause change in proximal measures. Generally, there is some evidence for better effects in guided than in unguided POI. Thus, a guided therapy may potentially increase the effects of iBA [49]; however, similar effects have been found between guided and unguided POI [50]. Taken together, the current evidence is not sufficient to support decisions regarding whether more redundancy, the repetition of the same content in more sessions, or a more complex, longer intervention with additional content is needed to affect depression.

Second, with assessments at 2-week intervals, the intervention or assessment interval was relatively short in comparison to the intervals of 6 to 17 weeks used in other study designs [23]. To allow more accurate conclusions about the treatment mechanisms, we chose this maximum-focused, one-module approach with a short interval with the aim of measuring rapid effects at the beginning of the treatment, which have also been reported in other clinical trials on depression, particularly with the use of antidepressants [51–53].

Although adherence is generally considered challenging in POIs, with completion rates as low as 80% and drop-out rates as high as 37% [5]. Although this study had better adherence, with completion rates of 85% at t_1_ and 76% at t_2_ and drop-out rates of 22% in the iBA group and 12% in the iMBI group, the 2-week intervention period was potentially too short for participants to use the intervention properly and transfer what they learned into everyday life. Indeed, 9 participants indicated that they did not access the modules at all (6/27, 22% in the iBA group and 3/25, 12% in the iMBI group). Unfortunately, we were not able to track user engagement and had to rely on self-report. Finally, we do not know if or how participants in the iBA group put the positive activities into practice. It is possible that a significant increase in physical activity is necessary for iBA to be effective in depression, as indicated by findings of an anti-inflammatory effect of BA with exercise on depression [51]. Hypothetically, a longer intervention interval in combination with automated support, such as email reminders, for the intervention as well as exercises (ie, physical activity) are important to explore whether the present, single-module iBA intervention or the intervention period and usage needs to be modified. Moreover, it would be helpful to assess the effects of engagement by monitoring the activities performed, to what degree physical activity was included, and how often each participant logged into the website and engaged with the content.

### Secondary Outcomes

Regarding the secondary outcomes, we found tentative support for the efficacy of the iBA. There was some support for the short-term superiority (ie, t_0_ to t_1_) of the iBA compared with the control groups based on the differences in behavior activation, as measured by the BADS, at trend level. This was further supported by the large effect sizes within the iBA group for change in activation (*d*=0.96-0.98). The post-hoc test showed that activation was greater with iBA than with iMBI. These differences, however, disappeared over the 4-week assessment interval. Surprisingly, the groups also differed at trend level with regard to change in mindfulness skills from t_0_ to t_1_, with a slightly higher improvement in the iBA group than in the iMBI group. However, the effect sizes were small and similar in the iBA and iMBI groups (d between 0.24 and 0.31). The changes in mindfulness, as assessed by the KIMS, in the iBA group may be considered surprising. However, Ly et al [28] also reported similar effects of BA and mindfulness in a measure of emotional acceptance/experiential avoidance.

Dysfunctional attitudes, as measured by the DAS-18B, decreased to a larger degree from t_0_ to t_2_ in the iBA group than in the control groups, particularly in comparison with the CAU group. This is in line with theoretical assumptions underlying CBT and results by Jacobsen et al [17] that show that change in activation leads to change in dysfunctional attitudes in depression. Because change in dysfunctional attitudes may also lead to change in depression in the long term, we consider that delayed effects on depressive symptoms in the iBA group are still plausible. However, due to the short study interval, we do not have any further follow-up data with which to verify this assumption.

Finally, we did not find group differences regarding an improvement in quality of life. This is unsurprising, considering the lack of difference in depression symptoms, which may be due to the short assessment interval.

### Mechanisms of Change

Although no treatment effect was found on the primary outcome, it is not only considered valid but highly important to study treatment processes such as mechanisms of change [45]. As planned, we entered the 3 potential treatment mechanisms in the mediation analysis: behavior activation, mindfulness, and dysfunctional attitudes. The only significant mediator for the effect of the intervention on PHQ-9 was behavior activation (BADS), further underlining the importance of behavior activation in the treatment of depression.

At first glance, this disagrees with the findings by Forand et al [52], who also used an online sample and highlighted the role of attitude modification. However, the intervention interval was much longer in their study (8 weeks vs 2 weeks), and it is still possible that effects will emerge at a later point in time in our sample. Moreover, the researchers used the Competencies Cognitive Therapy Scale to measure dysfunctional attitudes, while we used the DAS-18B, and the scales may not correlate. Generally, our results correspond with theoretical assumptions and evidence suggesting a link between an increase in the BADS and decrease in depressive symptoms [22]. Still, we did not find the expected treatment effect of the experimental intervention (iBA). Findings of the mediation analyses may thus not allow conclusions about the mechanisms of iBA, but they may be relevant to a general understanding of change in depression. We measured several other potential mechanisms (DAS-18B and KIMS) and concurrently considered them in the mediation model. Importantly, and as expected, short-term changes in the DAS-18B and KIMS did not mediate the treatment effects in this study. However, other mechanisms of change (eg, change in rumination) may play an important role in the treatment of depression, but these were not investigated in this study in order to minimize the burden on participants.

Additional mechanisms of change should be assessed in future studies. The BA model extends to possible mechanisms such as reinforcement, mood, and avoidance behavior [22]. This suggests that the relationship between behavior activation and depression is mediated by an initial increase in positive reinforcement, as found with undergraduate students [53]. These aspects of the BA theory should be considered in future studies.

### General Limitations

Some general limitations need to be considered. Most importantly, we did not verify the diagnostic criteria for major depression disorder in the sample. However, as the PHQ-9 has excellent psychometric properties and the cut-off of >4 is established in the literature, a change in the results is unlikely if a structured interview were to be included. Also, depressive symptoms were rather mild in the current sample, with means between 10.46 and 12.50 at baseline, and fluctuating symptoms and spontaneous remission are frequent in depression, with the clinical consequence that guidelines on MDD suggest “watchful waiting” as the first-line management for mild depression [54,55]. Also, the two modules (iBA and iMBI) differed in content; iMBI included an audio file (mindfulness exercise). This may have produced additional and unnecessary variance between interventions and should be controlled for in future dismantling studies. Moreover, the current sample was quite experienced in psychological interventions for depression, and activating or mindfulness treatment elements may have already been experienced by the participants in previous therapies and thus might not have the same effects as in treatment-naïve patients with MDD (see Table 1). The increasing number of available online interventions for psychological disorders may affect potential treatment samples for POIs. Expectations of the efficacy of a POI may generally be low if the participant had not benefited from an intervention before or had relapsed again after an intervention, ultimately leading to the self-fulfilling prophecy that POIs do not help. This is also reflected by evidence showing that attitudes toward online interventions and expectations moderate treatment outcome [56,57]. In addition, more than 75% of the participants were female, potentially compromising the generalizability of the findings. However, as depression occurs twice as often in women than in men [58], it seems particularly necessary to evaluate interventions in women. Finally, due to recruitment difficulties with the initial study protocol involving the use of the program in an outpatient clinic to bridge waiting times, changes in the trial protocol with regard to recruitment sources were unavoidable, and this also needs to be considered.

### Conclusion

To conclude, we did not find any evidence for the short-term efficacy of the iBA intervention on depression in treatment-experienced participants with mild to moderate levels of depressive symptoms. However, in comparison to the pooled control group, iBA was more effective with regard to the secondary outcomes, specifically an increase in activity over 2 weeks and a decrease in dysfunctional attitudes over 4 weeks. Furthermore, we found some support for short-term behavior activation being an important mediator for change in depression. Additional studies on brief interventions are needed to assess the mechanisms of change in POIs. It would also be helpful to test whether using longer intervention intervals, including automated support such as reminders to use the interventions or human support through a guided intervention enhances the treatment effects of iBA.

## Appendix

Multimedia Appendix 1 CONSORT eHEALTH checklist V1.6.1

## Abbreviations

BA: behavior activation.
BADS: Behavioral Activation for Depression Scale.
CAU: care as usual.
CBT: cognitive behavioral therapy.
CC: complete cases.
DAS: Dysfunctional Attitude Scale.
iBA: internet-based behavioral activation.
iMBI: internet-based mindfulness intervention.
ITT: intention-to-treat.
KIMS: Kentucky Inventory of Mindfulness Skills.
MDD: Major depressive disorder.
MBCT: mindfulness-based cognitive therapy.
POI: psychological online intervention.
WHOQOL: World Health Organization Quality of Life
